# Bond strength of cemented fiber posts to teeth with simulated internal root resorption

**DOI:** 10.4317/jced.56568

**Published:** 2020-03-01

**Authors:** Ricardo-Toledo Abreu, Jaiane-Bandoli Monteiro, Amjad Abu-Hasna, Thaís-da Silva-Alves Santos, Amanda-Maria-de Oliveira Dal Piva, Cesar-Rogerio Pucci, Carlos-Rocha-Gomes Torres, Cláudio-Antonio-Talge Carvalho

**Affiliations:** 1 São Paulo State University (Unesp), Department of Dental Materials and Prosthodontics, Department of Restorative Dentistry, 777 Eng. Francisco José Longo Avenue. São José dos Campos, SP

## Abstract

**Background:**

Teeth with internal root resorption (IRR) have guarded prognosis, considering that IRR defect could influence on the post bond strength. The aim of this study was to evaluate the bond strength and the bond interface between different glass fiber-reinforced posts (FRP) after cementation in teeth with simulated internal root resorption (IRR).

**Material and Methods:**

Forty-five (45) human premolar roots with simulated IRR were embedded in acrylic resin blocks and cross-sectioned into two segments, enabling them to be re-approximated by screws. Intracanal medication was inserted for 15-days, removed by passive ultrasonic irrigation (PUI) and examined by stereomicroscopy. The push-out bond strength of two fiber reinforced composite posts (Rebilda Post - RP) and Rebilda Post GT – GT, (VOCO) were evaluated at the cervical and IRR regions (n = 20). And, the bonded interface between resin cement and root dentine was analysed by scanning electron microscopy (SEM).

**Results:**

62.5% of IRR were not completely cleaned by PUI. Bond strength values at the cervical region (9.8 and 14.6 MPa) were higher than the IRR region (6.3 and 4.2 MPa). Micrographies showed bubbles in the cement and spaces in the bonded interface.

**Conclusions:**

RP post showed better bond strength at the cervical region while GT had better bond strength at the IRR region.

** Key words:**Endodontics, root canal filling materials, root resorption, X-Ray microtomography.

## Introduction

Root canal treatment is the indicated approach for internal root resorption (IRR) when the affected teeth is restorable and have a reasonable prognosis (1). Many cases have reported successful treatment for anterior and posterior teeth with radicular perforations and communication with the periodontal tissues (2-5). The IRR promotes defects that usually renders the unachievement of a complete bio-mechanical preparation; since, some parts of the lesion remain untouched during canal instrumentation and require adjunctive chemical debridement techniques, such as, passive ultrasonic irrigation (PUI) to improve the effectiveness of the root canal irrigants (6).

Structurally, the compromised teeth are susceptible to fail because the fracture strength is reduced since the circumferential canal wall thickness is decreased (7). For these compromised teeth, the best restorative materials should present elastic modulus similar to dentine, to create homogeneous stress distribution and improved fracture strength (8), as well as, to reduce the incidence of restoration failure as catastrophic fracture (9,10).

Fiber-reinforced posts (FRP) have fracture strength which is not influenced by post fit or post length (11,12). Restorations with intraradicular post aims to avoid unnecessary dentin wear thus root weakening (11). The tooth preparations are performed only to guarantee sufficient space for the post with minimal cement layer thickness, decreasing the occurance of adhesive failures (13). However, for tooth with compromised internal resorptive defects, they should be critically evaluated prior to the post insertion (10); because the greater the amount of remaining healthy tooth structure, the better the treatment prognosis (14).

The long-term success for restorations retained by FRP depends on factors related to their design (12) and to the attachment mechanism of adhesive systems applied to the intraradicular dentine (15). The predominant type of FRP failure is the adhesive failure (16,17). More recently, the anatomization of fiber posts was advocated to reduce the cement layer thickness (18), making it more uniform, void-free and improving the post retention and the bond strength between FRP and dentin (19,20). Conversely, a thick resin cement layer is generated when a post does not fit well in the root canal. This may result in the incorporation of air bubbles, which act as stress raisers during functioning (21). Based on this information, clinical questions erase considering if the IRR defect could influence on the post bond strength. Therefore, the aim of this present study was to evaluate the bond strength of different FRPs to dentin, in the presence and absence of simulated IRR defect. The potential interference of calcium hydroxide (Ca(OH)2) paste residues in the FRP bond strength was considered by simulating the removal of this medication prior to the FRP instalation. The null hypothesis was that there would be no influence of FRP type and root segment on the bond strength between FRP and dentin.

## Material and Methods

-Specimen preparation

Forty-five (45) human premolars with single-root were selected based on the dimensional and morphological similarities. The specimens were obtained from patients with the teeth destined for extraction, who gave consent to their use in the study. The present study was conducted in compliance with the Helsinki declaration for research on human subjects. The study protocol was approved by the Ethics and Research Committee at (São Paulo State University, Unesp/SJC), Institute of Science and Technology, Brazil) with the Project number 2.494.487.

The crowns were cross-sectioned at the cement-enamel junction using a carborundum disc (Dentorium, New York, USA) under water cooling. The root length was standardized at 16±0.5 mm. All root canals were instrumented with size 10 K-files (Dentsply Sirona, Petrópolis, RJ, Brazil) to achieve patency. Working length was determined by subtracting 1 mm from the root length. The canal space was irrigated with 3 mL of 1% sodium hypochlorite (NaOCl). The roots were embedded in cylinders (3 cm diameter; 2.5 cm high) of self-cured acrylic resin (TDV, Santa Catarina, Brazil) moulded in silicone (Classic Dental Articles, São Paulo, Brazil). Three holes were made in the acrylic resin cylinder with an electrical drill (3.96 mm diameter), approximately 3 mm apart and parallel to the longitudinal axis of the root, forming a triangle. The root included in acrylic resin was sectioned (8 mm from the cervical surface) perpendicularly to its longitudinal axis, using a precision cutting machine (Isomet 1000, Buehler, Illinois, USA), to obtain an upper portion and a lower portion. Three cylindrical screws (3.5 mm diameter) were positioned in the holes and fixed with threads to separate the set and return the two separated parts to their original position when necessary. For each specimen, a cavity was prepared for only one calibrated researcher with a diamond spherical drill (#3030, KG Sorensen, São Paulo, Brazil), in both halves (upper and lower) of the same tooth to simulate an IRR defect with standardized dimensions of 1.25 mm deep and 2.5 mm diameter.

-Chemomechanical debridement

The canals were prepared using Reciproc files R 50.05 (VDW, Munich, Germany) and irrigated with 5 mL of 2.5% NaOCl after the use of each file. Final irrigation was performed using 17% ethylenediaminetetraacetic acid (EDTA) (Biodinâmica, Paraná, Brazil) for 3 min, with the irrigant being activated with a size 50 K-file. Each canal was subsequently rinsed with 10 mL of sterile saline and dried with absorbent paper points (Dentsply Maillefer, Ballaigues, Switzerland). The canals were filled with Ca(OH)2 paste (UltraCal XS, Ultradent, Utah, USA), sealed with glass ionomer cement (Maxxion R, FGM, Santa Catarina, Brazil) and stored at 37 °C in deionised water for 15 days.

The glass ionomer cement temporary restoration was removed and the canals were irrigated with 5 mL of 2.5% NaOCl that was ultrasonically activated using an ultrasonic tip (E1; Irrisonic/Helse Ultrasonic, Brazil) mounted in a piezoelectric ultrasonic unit (Soni II, Ortus, Paraná, Brazil). The tip was placed 1 mm prior to the working length and activated for 1 min. The canals were filled again with EDTA 17%, which was also activated by the ultrasonic tip for 1 min.

The upper and lower portions of all specimens were examined at 10× and 30× magnification by optical stereomicroscopy (Discovery V20, Carl Zeiss, Gottingen, Germany). Three blind evaluators marked each micrograph according to the classification described in a previous study (6). The scores were: (0) the IRR cavity showed no debris; (1) less than half of the IRR cavity was filled with debris; (2) more than half of the IRR cavity was filled with debris; and (3) the IRR cavity was completely filled with debris.

-Fiber post cementation

The upper and lower portions of each specimen were bonded with cyanoacrylate glue (Super Bonder, Loctite, São Paulo, Brazil) at the inner edges of the acrylic resin of each portion, guided by the position of the screws for re-approximation of the root to its original position. The canals were obturated with size 50, 0.05 taper gutta-percha cone (Dentsply Maillefer, Ballaigues, Switzerland) and AH Plus root canal sealer (Dentsply Maillefer). After complete setting of the sealer (24 h), the post space was prepared in each filled canal using size I-III post-drills (Dentsply Maillefer). Each drill was length-controlled to 10 mm from the cervical third of the root. Post size was determined after examination of the canal thickness.

The canals were divided according to the post type: conventional FRP (Rebilda Posts -RP, VOCO, Cuxhaven, Germany) (n=21) and FRP composed of various fine individual posts (0.3 mm diameter) (Rebilda Posts GT - GT; VOCO) (n=24). One specimen from each group was used to analyse the bonded interface between the resin cement and dentin. And, three specimens from GT group were used for micro-computed tomography (micro-CT) investigation.

All posts were cleaned with 92.8% ethanol, rinsed with distilled water and dried with oil-free air. Then, a silane (Ceramic Bond; VOCO) layer was actively applied on the post surface for 20 s, left undisturbed for 60 s and dried with oil-free air. Each post space was irrigated with 92.8%, ethanol, rinsed with distilled water and dried with absorbent paper points. A dual-cured adhesive Futurabond U, (VOCO) was actively applied in the canal for 20 s. Rebilda DC resin cement (VOCO) was used for cementation of both groups. The resin cement was applied directly to the root canal using an intracanal tip adapted to a QuickMix syringe (VOCO).

The posts were inserted into the resin cement-filled post space using finger pressure. Each post was cut at the location of the length control rubber stopper with a diamond bur without water cooling, according to the manufacturer’s instruction. Both groups were light-cured at the cervical end for 40 s with a light-emitting-diode light-curing unit (Valo, Ultradent Products Inc., South Jordan, Utah, USA) with an output intensity of 1000 mW/cm2 at 395-480 nm.

-Push-out bond strength

Perpendicular cuts were made with a precision cutting machine (Isomet 1000, Buehler, Illinois, USA) under water cooling, at distances of 4 mm and 8 mm from the cervical root level to obtain root slices (1.0 ± 0.1 mm thick). Push-out bond strength was performed with a universal testing machine (EMIC DL 1000, Paraná, Brazil) at a cross-head speed of 1 mm/min, using a 50 kgf load cell, until failure. During the mechanical test, each slice was placed on a metallic device with a 3 mm diameter central opening, which was larger than the canal diameter. The smaller canal diameter side of each root slice was placed against the indenter. The load was applied to the center of the slice from the smallest to the largest diameter.

The maximum load at failure (in Newtons) was divided by the adhesion area (mm²) of the bonded interface to generate the push-out bond strength. The adhesion area of the root canal material was calculated by using the formula: πh(R1+R2), where h = root slice height, R1 and R2 = radius of the major and minor width of the canal space.

-Failure mode and statistical analysis

Qualitative analysis was performed with stereomicroscopy (Discovery V20, Germany) at 30× magnification to evaluate the type of failure of each specimen. Failure mode was classified as: ACD) adhesive failure between resin cement and dentine; (ACP) adhesive failure between resin cement and post; (M) mixed failure between resin cement and dentine or between resin cement and post; (CC) cohesive failure of cement; and (CD) cohesive failure of dentine.

Bond strength data were statistically analysed using the Kruskal-Wallis, Mann Whitney and Dunn’s multiple-comparison test at 5% significance level. The data were statistically analysed with Minitab software (Minitab 17 for Windows, 2004). Representative fractured specimens were inspected under Scanning Electron Microscopy (SEM) at 50 and 100x magnification.

-Analysis of the bond interface

SEM was performed to analyse the bonded interface between the resin cement and root dentine. Thus, a representative specimen from each group was prepared and cut along the longitudinal axis. Phosphoric acid (37%; Condac 37, FGM, Campo Mourão, Brazil) was applied superficially for 30 s and the specimens were immersed in 17% EDTA for another 5 min. The specimens were dehydrated in increasing concentrations of ethanol (70, 80 and 90%) for 30 min each, followed by 100% isopropyl alcohol twice for 30 min each. The specimens were placed on aluminum stubs, sputter-coated with gold/palladium (Emitech SC7620, Quorum Technologies Ltda., Laughton, UK), and examined with a SEM (Inspect S50, FEI Company, Brno, Czech Republic) at 2000× and 4000× magnification.

-Micro-CT and 3D reconstruction 

Three specimens from GT group were evaluated by micro-CT (Skyscan model 1176, Kontich, Belgium) to investigate the disposition of the posts, cementation line defects and possible interference of remnants during the root canal treatment. The scanning was performed with an X-ray source of 50 kV and 497 μA, a 0.5 mm thick aluminum filter, 18 μm pixel size, 180º tube rotation, 0.8º rotation step, 4 s exposure time and 10 min scanning time. And, the reconstruction of the 3D root models was performed with the CTan software tool and subsequently visualised using the CTvox software (version 1.8.1.5, Skyscan, Belgium).

## Results

-Cleanliness of IRR

Scores (percentages) reflecting the cleanliness of the IRR defect space after irrigant agitation with PUI are summarised in Table 1. The PUI technique was not able to completely remove the Ca(OH)2 from the IRR in most of the specimens (62.5%).

**Table 1:**
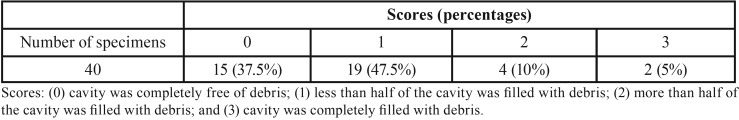
Cleanliness of the simulated internal root resorption (IRR) cavity after the passive ultrasonic irrigation (PUI) technique.

-Push-out bond strength and failure modes

The descriptive data is presented in Table 2 shows the mean bond strength values, standard deviations and coefficients of data variation derived from the RP and GT groups cemented at the cervical and IRR levels. Specimens with root dentine cohesive failure were excluded from the statistical analysis. RP bond strength mean value at the cervical region was higher (14.8 ± 5.7 MPa) and statistically different from the RP bond strength at the IRR defect area (4.2 ± 4.7 MPa). When both posts were compared in the IRR region, it was possible to observe that GT post presented higher bond strength value (6.2 ± 5.1 MPa) compared with RP post (4.2 ± 4.7 MPa). However, there was no statistical difference between them.

**Table 2:**
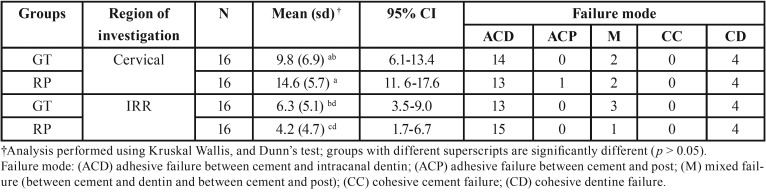
Means (MPa), standard deviation (sd) and confidence intervals of bond strength at the cervical and IRR regions and failure mode analysis.

**Figure 1:**
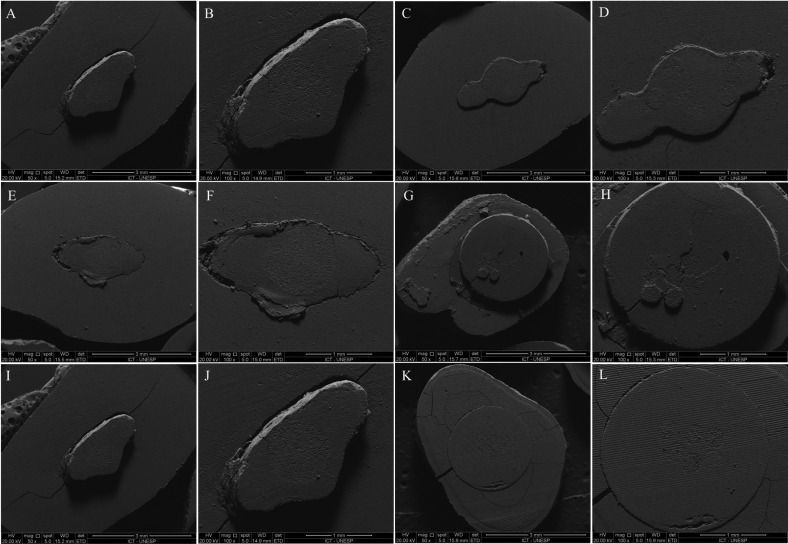
Representative SEM images of failure modes of the debonded Rebilda Post (left) and Rebilda Post GT (right) at 50× and 100× magnifications. A-F and I-J. Adhesive failure between cement and root dentin. G-H. Mixed failure between cement and dentin and between cement and post. K-L. Cohesive failure of root dentin.

Figure 1 shows SEM images of the predominant type of failures observed after the push-out test. The predominant failures were adhesive failure between resin cement and dentine, mixed failure and cohesive dentine failure.

-Bond interface

**Figure 2:**
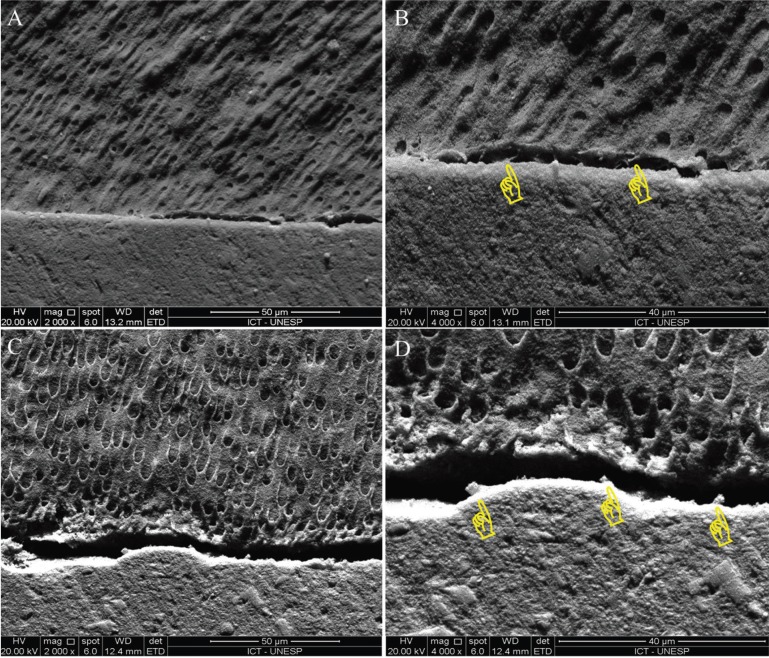
Representative SEM micrographs of the bond interface for the RP (A,B) and GT (C,D) posts at 2000× (left) and 4000× (right) magnification. Yellow pointers indicate the presence of resin tags.

Figure 2 contains SEM micrographies of the bond interface between resin cement and dentin. For both groups, resin tags extended minimally into the dentinal tubules. True interfacial gaps or artefactual separation of the bonded interface, not distinguishable from one to another because of the specimen desiccation using a high vacuum, could be discerned in both groups.

-Micro-CT and 3D reconstruction 

**Figure 3:**
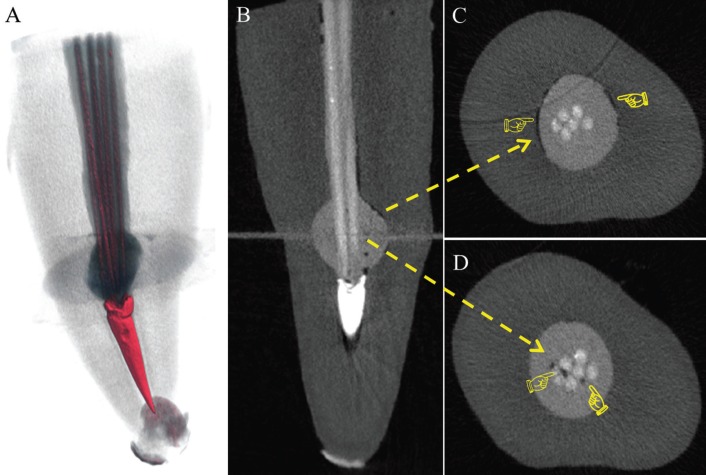
Micro-CT analysis of a representative specimen from GT group (pixel size = 18 μm) showing the 3D volumes of voids after the root canal treatment.

Figure 3 shows a representative specimen from GT group. Failures were identified at bond interface between the resin cement and root dentine. Under axial and longitudinal sections it was possible to identify failures in the resin cement as unfilled spaces and bubbles in root canal, mainly in isthmus regions and, in larger numbers within the IRR simulated defect.

## Discussion

Internal root resorption (IRR) produces an irregular increase in the pulp internal space because of the active process of inflammation (22) which may be stabilised using contemporary root canal treatment and restorative techniques (5). The use of fiber-reinforced posts (FRP) represents one of the restorative options (23) to rehabilitate tooth with IRR defect. For the preservation of tooth structure, different FRPs were developed (24), such as the GT system investigated in the present study. The use of these posts in the root canal may be advantageous for the maintenance of a satisfactory amount of intact dentine. It’s not necessary to widen the root canal during the desobturation. As a new material, GT post still has some questions that have not been answered in the literature, such as if its use could influence on the integrity of the bonded interface between the resin cement and root dentine after cementation.

The clinical survival of a fiber post is affected by many factors. Therefore, the clinician should be astute in the selection of a material that best suits the individual needs of each tooth (25). According to the manufacturer, GT consists of a bundle of thin translucent individual posts (diameter of each post = 0.3 mm), and is indicated for atypical root canal anatomy and pronounced taper. The push-out test for FRPs in the literature suggests the use of a centralised axial force over the cemented post. Hence, the GT posts do not allow the application of a centralised force over the post, but over the cement-post combination instead. Therefore, GT group presented higher bond strength at the simulated IRR level compared with the group cemented with RP posts. These results may be related to the scattering action of the individual posts within the GT system at the simulated IRR level. The IRR is highly frequent in the root middle third (26), with diameters up to 1.6 mm diameter and depth of 0.8 mm (27). Clinically, the IRR process creates irregular lesions (28). In this study, the simulation of a IRR defect was performed using a spherical drill to obtain standardized cavities in the middle third of the root, with diameter and thickness similar to the data reported in the literature (29).

Since the ultrasonic agitation increases the penetration of irrigants into the dentinal tubules, PUI is generally perceived to be an effective technique in removing intracanal smear layer and debris (6). In another study, PUI completely removed the Ca(OH)2 paste in only 44.4% of the IRR cavities (28). Due to the dimensions of the simulated IRR cavities were standardised with diamond drill, all had regular borders compared to the natural irregular IRR lesions. Therefore, the removal of the intracanal medicament should be considerably easier. Nevertheless, complete cleaning was only identified in 37.5% of the simulated IRR cavities in the present study.

The PUI protocol tested in this study was not able to completely clean the intracanal medicament remnants from most of the specimens (62.5%). Of note, PUI of the entire canal space additionally with 17% EDTA and final irrigation with saline were performed prior to the post cementation. In clinical practice, this would translate the potential interference with post cementation and consequent decrease the cemented post bond strength to the root dentin (30). 

Because bond strength of post segments at the simulated IRR level was lower than the cervical level, the null hypothesis has to be rejected. Post bond strength to dentin may be affected by the post location inside the root canal. In the cervical third of the canal space, the strength is higher than the middle third regardless of the type of cement employed (19,31,32). The reason for the lower bond strength values at the IRR level may be attributed to the greater penetration distance of the light employed for photopolymerisation (33), and to susceptibility of creating air voids in the presence of thick cement layers (21,24). In addition, thicker cement layer is related to higher stress concentration due to higher polymerization shrinkage (19). 

With respect to the post conformation, it has been reported that the bond strength is influenced by the adaptation of the post to the root canal; lower cement thickness results in better bond strength (13,19,34). It should be noted that the bond strength mean values for RP were higher than GT at the cervical level. This may be interpreted as conventional post systems adapt better when there is less cement volume and higher mechanical strength generated by friction between the post and the canal wall (20). Because the bond strength of different post systems and canal region (cervical or IRR level) were different, the null hypothesis was rejected.

Failure mode evaluation is helpful in interpreting the bond strength results. An earlier study investigated the effects of various resin cements on the bond strength between post and dentin and showed that most of the found defects occurred between the resin cement and the root dentine (31). In the present study, the type of fiber post did not appear to have interfered with the micromechanical bonding process when the same adhesive system was applied to the intraradicular dentine (32). This was probably happened because the higher cement thickness around the posts in all root canal.

Different variables may have an impact on the adhesion of the resin cement to a fiber post or to a dentine substrate during post dislodgement. These variables include composition (31,32) and the viscosity of the dentine adhesive and the resin cement (21,36). The intraradicular dentine of the specimens was treated with Futurabond U primer, which contains organic acids, dimethacrylate, amines and butylhydroxytoluene. The posts were cemented with dual-cured Rebilda DC resin cement (composition: bisphenol A glycidyl methacrylate, diurethane dimethylmethacrylate, butylhydroxytoluene and benzoyl peroxide). The methacrylate is responsible for the hydrophobic behaviour, viscosity and the mechanical properties of the resin cement system (37). The low viscosity of diurethane dimethylmethacrylate contributes to the flow of the resin cement (38), however, the decrease in viscosity has the potential to increase the incorporation of air voids during mixing. With the use of micro-CT, air voids could be identified in the resin cement, especially within the simulated IRR cavity.

Another controversy regarding the GT posts is the adequacy in silanization of the individual internal portions of each miniature post component. One cannot not guarantee that this silanization occurred uniformly and effectively by following the manufacturer’s recommendations. Taking into account the absence of failure between individual miniature post component and the resin cement, it is speculated that inadequate silanization is not responsible for the reduced bond strength at the IRR level. This speculation is supported by the findings of a previous study, where the authors did not observe differences in the silanization procedure for the fiber post adhesion (39).

## Conclusions

Within the limitations of the present study, it may be concluded that the adhesive bond strength between post and dentin is influenced by the presence of IRR defect. In addition, air voids are abundant in the presence of a thick, uneven layer of resin cement. These issues have to be considered by clinicians in their clinical use of the canal space for retention of resin composite cores when internal resorption is present in the root canal system.
